# Hiv infected patients in intensive care: an observational study of admissions between 1996-2006 and 2007-2014

**DOI:** 10.1186/2197-425X-3-S1-A644

**Published:** 2015-10-01

**Authors:** J Highgate, A Curtis, F Baldwin, Y Gilleece

**Affiliations:** Anaesthetics & Intensive Care Medicine, Brighton and Sussex University Hospitals Trust, Brighton, United Kingdom; HIV & GUM Medicine, Brighton and Sussex University Hospitals Trust, Brighton, United Kingdom

## Introduction

With the advent of widely available HIV testing and the introduction of antiretroviral agents, life expectancy of HIV infected patients has increased. Historically patients with HIV requiring ITU admission had a poor outcome, with mortality up to 70% however in recent years ITU mortality rates have fallen ([1]). An aging population presenting with both HIV complications and non-HIV related disease combined with improved survival rates has implications for ITU management and prognostication.

## Objectives

To undertake a retrospective observational study comparing the clinical presentation of patients with confirmed HIV infection admitted to the intensive care unit at the Royal Sussex County Hospital, Brighton between 1996 - 2006 and 2007 - 2014.

## Methods

Patients were identified from the ITU electronic records using either the entry of a diagnosis of HIV or a CD4 count. Diagnosis was confirmed using pathology and HIV department patient records. Data including demographics and level of organ support was collected. Comparison was made to data available from 1996 - 2006.

## Results

Between 2007-14, 75 patients were admitted to the ITU with a total of 86 patient episodes; an increase from 48 admissions in the previous period. The mean ITU length of stay was 6.1 days (range < 1 - 37 days). The patient group was largely male with a significant increase in the mean male age since 2007 (50.7 vs. 41.6, p = 0.0002). While the diagnosis of HIV was made in 24% of patients during the hospital admission there was a statistically significant increase in patients presenting to ITU established on antiretroviral therapy (48 vs. 17, p = 0.0096) and an associated increase in non-HIV related disease leading to admission. Between the comparison periods, there was no statistically significant difference in the number of patients receiving respiratory support (invasive or non-invasive ventilation, 58 vs. 38, p = 0.20) or renal replacement therapy (6 vs. 5, p = 0.20). There was no correlation between ITU mortality and either CD4 count (p = 0.52) or viral load (p = 0.85). Mortality rates halved between the comparison groups (10% vs. 20%) with no statistically significant difference between mortality in HIV-infected patients and the general ITU population since 2007 (10% vs. 17.7%, p = 0.39).

## Conclusions

ITU admissions in HIV infected patients have increased since 1996. Testing of patients for HIV remains important as undiagnosed patients continue to present however increasing numbers of patients are admitted on antiretroviral therapy with non-HIV related disease. The level of respiratory and renal support has remained constant but mortality rates have halved. With increasing survival and no correlation between mortality and CD4 count or viral load, all patients should be considered for admission.

## References

[1] Coquet (2010). Survival trends in critically ill HIV-infected patients in the highly active antiretroviral therapy era. Critical Care.

